# Integrating Nutrition and Exercise to Mitigate Cardiometabolic Risk and Enhance Outcomes in Lung Cancer During the Era of Immunotherapy and Targeted Therapy

**DOI:** 10.3390/nu18142290

**Published:** 2026-07-13

**Authors:** Giuseppina Gallucci, Alessandro Inno, Stefania Fugazzaro, Stefania Costi, Silvia Di Leo, Debora Pezzuolo, Francesca Zanelli, Patrizia Ciammella, Alessandro Navazio, Carmine Pinto, Luigi Tarantini

**Affiliations:** 1 Independent Researcher, 85025 Melfi, Italy; 2 Medical Oncology, IRCCS Ospedale Sacro Cuore Don Calabria, 37024 Negrar di Valpolicella, Italy; alessandro.inno@sacrocuore.it; 3 Physical Medicine and Rehabilitation Unit, Azienda Unità Sanitaria Locale (AUSL)-IRCCS di Reggio Emilia, 42122 Reggio Emilia, Italy; stefania.fugazzaro@ausl.re.it; 4 Research and EBP Unit, Health Professions Department, Azienda Unità Sanitaria Locale (AUSL)-IRCCS di Reggio Emilia, 42122 Reggio Emilia, Italy; stefania.costi2@ausl.re.it; 5 Department of Surgery, Medicine, Dentistry and Morphological Sciences, University of Modena and Reggio Emilia, 41124 Modena, Italy; 6 Psycho-Oncology Unit, Azienda Unità Sanitaria Locale (AUSL)-IRCCS di Reggio Emilia, 42122 Reggio Emilia, Italy; silvia.dileo@ausl.re.it; 7 Medicina Oncologica, Ospedale di Guastalla e Correggio, Area Nord, Azienda Unità Sanitaria Locale (AUSL)-IRCCS di Reggio Emilia, 42122 Reggio Emilia, Italy; debora.pezzuolo@ausl.re.it; 8 Provincial Medical Oncology, Department of Oncology and Advanced Technologies, Azienda Unità Sanitaria Locale (AUSL)-IRCCS di Reggio Emilia, 42122 Reggio Emilia, Italy; francesca.zanelli@ausl.re.it (F.Z.); carmine.pinto@ausl.re.it (C.P.); 9 Radiation Oncology Unit, Azienda Unità Sanitaria Locale (AUSL)-IRCCS di Reggio Emilia, 42122 Reggio Emilia, Italy; 10 Cardioncology Clinic—Cardiologia Ospedaliera, Azienda Unità Sanitaria Locale (AUSL)-IRCCS di Reggio Emilia, 42122 Reggio Emilia, Italy; alessandro.navazio@ausl.re.it (A.N.); luigi.tarantini@ausl.re.it (L.T.)

**Keywords:** lung cancer, nutrition, exercise, cardiac metabolism, sarcopenia, malnutrition, cardiovascular risk, therapy response, lifestyle intervention, inflammation, immune system, immunotherapy, targeted therapy

## Abstract

Over the last few decades, survival among patients with lung cancer (LC) has progressively improved due to major advances in treatment strategies, particularly the introduction of immunotherapy and targeted therapies, as well as the increased detection of early-stage disease resulting from the widespread use of chest computed tomography (CT). Although the reduction in mortality, frequently achieved through effective control of the primary disease, represents a major therapeutic success, it also raises new clinical challenges, including the long-term management of cancer remission or disease stability and the competing risk of adverse outcomes related to comorbidities and treatment-related toxicities. Among these, cardiovascular (CV) complications have emerged as particularly relevant because of their frequency and prognostic impact. Within the framework of a holistic long-term management approach, increasing attention should be directed toward non-pharmacological interventions targeting lifestyle factors, particularly nutrition and physical exercise, whose role remains underestimated. These interventions may modulate chronic inflammation and immune responses, which are key drivers influencing both the effectiveness of novel anticancer therapies and the progression of cardiovascular complications. Patients with LC frequently present malnutrition and unfavorable lifestyle patterns associated with substantial physical and psychological stress, factors that may negatively affect treatment outcomes and overall prognosis. This narrative review examines the emerging role of targeted nutritional strategies and structured physical exercise as integral components of supportive care in LC, with a specific focus on their impact on cardiac metabolism, CV risk, and response to anticancer therapies, including immunotherapy.

## 1. Introduction

Lung cancer (LC), one of the leading causes of cancer-related mortality worldwide [1], is typically diagnosed at a median age of approximately 70 years, a stage of life characterized by a high prevalence of multimorbidity [2,3,4]. In addition, patients with LC frequently present unhealthy lifestyle factors [5,6] particularly smoking [7], which further contribute to the development of age- and lifestyle-related cardiovascular risk factors [8,9,10]. As a result, LC is frequently complicated by pre-existing cardiovascular disease (CVD), which may also be exacerbated by anticancer treatments [11].

CVD is particularly relevant, affecting 30–50% of cases [12,13] and contributing to increased mortality (~30%) when coexisting with LC [14]. CVD is the second leading cause of death in LC patients, following cancer progression [15], and its impact on treatment decisions is well known [16]. The strong bidirectional relationship between LC and CVD is indeed documented by the increasing trend of CVD-related hospitalizations among LC patients, the higher incidence of LC in individuals with pre-existing CVD [17,18] and the shared risk factors, such as smoking and airborne environmental contaminants (AECs) [7,19]. Moreover, lifestyle-related CV risk factors, such as physical inactivity and unhealthy diet, are well-known contributors to LC risk [5,6].

Beyond sharing common risk factors, cancer and cardiovascular disease (CVD) also exhibit several overlapping biological mechanisms that may contribute to their bidirectional relationship [20,21,22]:

- Endothelial dysfunction: loss of endothelial integrity impairs the function of endothelial cells, which act as key sensors of hemodynamic forces and play a central role in vascular homeostasis, resilience, and adaptation to environmental stressors (exposome).
- Chronic inflammation: persistent inflammatory activation promotes immune dysregulation, contributing to the development of several noncommunicable diseases, including CVD and cancer, supporting the concept of a common pathogenic soil.
- Cellular proliferation: activation of mitogenic pathways can promote uncontrolled cell growth as well as cardiovascular remodeling and cardiac fibrosis.
- Resistance to cell death: dysregulation of cellular stress responses and apoptotic pathways favors cell survival under adverse conditions.
- Neurohormonal activation: increased levels of cardiovascular neurohormones play a pivotal role in acute and chronic heart failure; however, similar mediators may also be produced by malignant cells within the tumor microenvironment.
- Angiogenesis: angiogenic pathways are involved in endothelial cell survival and contribute to tumor growth, invasion, and metastatic spread.
- Genomic instability: genomic alterations, including clonal hematopoiesis of indeterminate potential (CHIP), have emerged as risk factors for CVD and may also be linked to chronic inflammatory and atherosclerotic processes through sustained stimulation of hematopoietic stem cell proliferation.
- Metabolic reprogramming: Both cardiomyocytes and cancer cells exhibit remarkable metabolic adaptability, enabling the preservation of cellular functions during stress conditions. Various stressors may disrupt the balance between ATP demand and oxidative metabolism. In the failing heart, adaptive responses include a shift from oxidative phosphorylation toward glycolytic ATP production, with specific metabolites such as ketone bodies potentially acting as epigenetic regulators. A similar metabolic phenotype is observed in cancer cells; however, whereas metabolic adaptation in the heart serves a compensatory and protective role, in tumors metabolic remodeling sustains malignant progression and phenotype maintenance.

Malnutrition [23], systemic inflammation, and sarcopenia [24] further exacerbate this vulnerability, contributing to cardiometabolic stress and increased risk of heart failure (HF), arrhythmias, and ischemic events [25,26].

Addressing these overlapping risks has become increasingly important considering the significant improvement in survival among patients with lung cancer (LC), driven by the implementation of screening programs and the introduction of immunotherapy and targeted therapies [27]. While these therapeutic advances have markedly improved prognosis, they have also shifted attention toward the long-term cardiometabolic consequences of treatment and the growing need for awareness of potential cardiotoxicity. To maximize the benefits of these therapies, a comprehensive baseline assessment of LC patients is essential to identify CV risk and implement preventive strategies aimed at reducing the burden of CVD.

Sedentary lifestyle and unhealthy dietary patterns are commonly observed both in individuals at increased risk of developing lung cancer and in patients already diagnosed with the disease.

In the UK Biobank cohort (416,588 participants, including 1782 LC cases), adherence to a dietary pattern characterized by high consumption of fruits, vegetables, wholegrains, and dietary fiber, together with low intake of red and processed meat, was associated with a reduced incidence of LC. Conversely, LC occurred more frequently among older men with lower socioeconomic status, poorer educational attainment, and higher rates of smoking and alcohol consumption [28]. Active smokers also commonly exhibit unfavorable lifestyle habits that further impair immuno-cardio-metabolic homeostasis [29,30].

Several mechanisms may explain the different effects of the so-called “Prudent” and Western” dietary patterns on LC risk. High consumption of red and processed meats may increase carcinogenic exposure through the endogenous formation of compounds such as N-nitroso compounds, while also promoting chronic inflammation and alterations in local immune responses. In contrast, fruits, vegetables, and wholegrains are rich in bioactive micronutrients and antioxidant compounds that may exert anti-inflammatory and protective effects. Among these, carotenoids such as β-carotene have been associated with reduced oxidative stress and inflammation related to smoking exposure. However, randomized trials evaluating vitamin A and β-carotene supplementation have failed to confirm these benefits and, in some populations of smokers, have even suggested potentially harmful effects [31]. Dietary fiber may represent an additional protective factor against LC. Several studies have suggested that the antitumor effects of fiber may be mediated by short-chain fatty acids generated by gut microbiota fermentation [32]. Preclinical evidence indicates that fermentable dietary fibers can modulate the pulmonary immune microenvironment through alterations in gut–lung axis signaling, promoting favorable changes in both gut and lung microbiota composition [33].

Tailored nutritional strategies and structured exercise interventions not only counteract cachexia and metabolic dysfunction but also preserve cardiac function, improve tolerance to anticancer therapy, and reduce the likelihood of competing CV events [34,35]. This review addresses non-pharmacological treatment to improve cardiovascular health of LC patients in order to create a less vulnerable population. It also tries to find converging pathways that are all intertwined and that may eventually meet at a molecular level, highlighting the importance of translation and clinical studies to identify all these converging patterns.

## 2. Metabolic and Cardiovascular Alterations in Lung Cancer

### 2.1. Systemic Inflammation and Cardiometabolic Risk

As Libby pointed out “inflammation is a common contributor to cancer, aging, and cardiovascular diseases” [36]. Major drivers of atherosclerosis are indeed age, low-density lipoprotein (LDL) cholesterol, hypertension, diabetes mellitus, smoking and obesity; many pathogenic processes (e.g., endothelial–mesenchymal transition, smooth muscle cell and macrophage proliferation, dysregulated cell death, defective efferocytosis, leukocyte infiltration, plaque angiogenesis, extracellular matrix remodeling, rupture and erosion) and numerous mediators such as fibroblast growth factor (FGF), matrix metalloproteinase (MMP), platelet-derived growth factor (PDGF), transforming growth factor beta (TGF-b) and vascular endothelial growth factor (VEGF) are implicated in both oncogenesis and atherogenesis [21]. The release of adipokines from the adipose tissue (an immunologically active organ) orchestrates metabolic inflammation that links obesity to cardiometabolic risk [37]. The dynamic inflammatory process of atherosclerotic plaque begins with high levels of oxidized LDLs and remnants of triglyceride-rich lipoproteins that gain access to the subendothelial space and elicit a danger signal that activates the NOD [nucleotide oligomerization domain]-containing, LRR [leucine-rich repeat]-containing, and PYD [pyrin domain]-containing protein3 (NLRP3) inflammasome [38,39] in innate immune cells. The activation of inflammasome induces a cascade of inflammatory cytokines such as interleukin 1 (IL-1) and interleukin 6 (IL-6) and upregulates high-sensitivity C-reactive protein (hs-CRP) in the liver eventually leading to atherosclerotic lesions that disrupt the integrity of endothelial cells (ECs) and their atheroprotective role (e.g., secretion of vasoactive substances affecting vasodilatation, platelet function, and monocyte infiltration). The activated ECs enhance the process of tissue inflammation by steering the recruited immune cells towards a proinflammatory phenotype [40,41]. Sustained stimulation of ECs may also be involved in the endothelial-to-mesenchymal transition that leads to fibrosis [42]. Immune cells (monocytes, macrophages, dendritic cells, neutrophils, T cells, and B cells) are deeply committed in the inflammatory scenario of atherosclerosis [43]. In LC, chronic inflammation, mediated by cytokines such as IL-6, tumor necrosis factor-α (TNF-α), and interleukin-1β (IL-1β), drives proteolysis, lipolysis, and mitochondrial dysfunction [44,45,46,47]. The key role of mitochondria in atherogenesis has been recently highlighted in a study that has determined the expression levels of mitochondrial DNA (mtDNA) in atherosclerotic lesions revealing a novel mechanism by which mtDNA arranges mitochondrial function-related gene expression in macrophages and promotes atherosclerosis [48]. All these effects of inflammation compromise skeletal [49,50] and cardiac muscle energetics [51,52] promote endothelial dysfunction [53] and CV morbidity [54], while the systemic inflammatory milieu directly contributes to sarcopenia, reduced exercise capacity, and decreased tolerance to anticancer therapy [55].

Cancer cells and cardiomyocytes use similar mechanisms of copying with stressors. Moreover, there is a crosstalk between cancer and the heart at a molecular level that increases CVD risk. As a consequence, CVDs are driven not only by cancer therapy-induced cardiotoxicity but also from the biology of cancer itself. Regulators of mitochondrial function are mutated in tumors and this mutation leads to increased production of oncometabolites [56]. In addition, epidemiologic data suggest a close link between social determinants of health (SDOH) and CVD [57]; systemic inflammation (defined by hs-CRP) and traditional cardiovascular–kidney–metabolic (CKM) risk (defined by the AHA PREVENT HF risk score) seem to underline the relationship between SDOH and incident HF and this association is likely independent of subclinical atherosclerosis and myocardial injury. The hypothesis is confirmed by a recent study among MESA cohort participants without HF at baseline in which inflammation and traditional CKM risk explained approximately one-third of the associations between SDOH and HF, thus conferring a key role to inflammation in the complex SDOH/HF relationship and emphasizing the need to address social/biological intersections to prevent HF in vulnerable populations [58]. The intersection of SDOH and CVD is strongly linked to excess stress hormones, inflammation, immune cell function and cellular aging [59]; from their interaction, a strong activation of the sympathetic nervous system and the hypothalamic–pituitary–adrenal axis is elicited with increasing amounts of inflammation [57].

### 2.2. Cancer Cachexia and Sarcopenia

Cancer cachexia, characterized by ongoing loss of skeletal muscle mass and function, is prevalent in up to 70% of patients with advanced lung cancer [60]. It is a dynamic process in which pre-cachexia represents a subclinical stage with widespread inflammation and a weight loss < 5%, cachexia emerges as a clinical stage with weight loss > 5% (reduced to >2% in presence of sarcopenia and BMI < 20) and advanced cachexia is present in advanced cancer refractory to anticancer treatment [61]. NSCLC is associated with severe forms of cachexia; the metabolic derangement in cancer cells is responsible for the remodeling in both skeletal and heart muscle [56]. Cancer-related inflammation affects the nutritional status and the combined effect of inflammation and cachexia contribute to immunosuppression with a subsequent negative impact on the antineoplastic immune response and immunotherapy efficacy [62,63]. Cancer-related cachexia includes sarcopenia, defined by reduced lean mass often accompanied by increased visceral and ectopic fat. Sarcopenia, identified by low muscle strength and low muscle mass [64], is found in ~50% of NSCLC patients [24] and is associated with chronic inflammation and accelerated CVD progression [65]. Baseline sarcopenia in patients scheduled for ICI therapy is linked to poorer treatment response [66,67], increased toxicity [68], higher risk of CV events [65] and shorter survival., highlighting the interplay between metabolic derangements and competing CV risk. Mechanisms include increased resting energy expenditure, hormonal dysregulation, mitochondrial impairment, and chronic inflammation [69,70]. It is important to identify patients in subclinical and early clinical stages to optimize the beneficial effect of non-pharmacological interventions.

### 2.3. Cancer Therapy-Induced Cardiac Metabolic Dysfunction

Chemotherapy [71,72], targeted therapy [11,73], radiotherapy [74] and immunotherapy [75,76,77,78,79,80,81] exacerbate cardiac metabolic stress via mitochondrial injury, oxidative stress, and impaired substrate utilization, eliciting the cancer treatment-induced metabolic syndrome (CTMS), a key driver of CV risk. Immune check point inhibitors (ICIs) and targeted therapies have indeed transformed the therapeutic landscape of NSCLC, but their use has been increasingly burdened by cardiotoxicity events among which ICI-associated myocarditis is the most dreadful complication. A recent paper by Power JR et al. [82] has defined ICI-associated myocarditis as a component of a generalized myotoxicity, which occurs in 0.3–1% of patients treated with ICI, but with a mortality of 30–35% documented in previous studies [83,84,85]. The authors found that a risk score incorporating magnitude of troponin increase, pre-existing, thymoma, low QRS voltage, depressed LVEF, and cardiomuscular symptoms was associated with myocarditis severity [82]. This chapter focuses on targeted therapy-associated metabolic complications. Targeted therapies for LC include epithelial growth factor receptor (EGFR) inhibitors, anaplastic lymphoma kinase/c-ros oncogene 1 (ALK/ROS1) inhibitors, V-Raf murine sarcoma viral oncogene homolog B/mitogen-activated extracellular signal-regulated kinase (BRAF/MEK) inhibitors, Rearranged during Transfection (RET) inhibitors, and vascular endothelial growth factor (VEGF) pathway agents. First-generation EGFR-I such as erlotinib and gefitinib are associated with ischemic events and hypomagnesemia-mediated cardiac dysfunction. Overall cardiac risk is lower in third-generation agents such as Osimertinib and Lazertinib [86,87]. Among ALK/ROS1 inhibitors, Lorlatinib is uniquely associated with pronounced hypercholesterolemia, hypertriglyceridemia, weight gain and hypertension. Grade 3–4 hypertriglyceridemia and hypercholesterolemia are common at 5-year follow up [88]. Alectinib causes visceral adiposity and sarcopenic obesity with a risk of metabolic syndrome in patients with substantial weight gain [89]. Selpercatinib, a RET inhibitor, is associated with hypertension [11]. As far as VEGF inhibitors are concerned, bevacizumab, ramucirumab and multi-kinase inhibitors (cabozantinib, lenvatinib) are associated with hypertension linked to reduced NO bioavailability, endothelin-1 upregulation and capillary rarefaction [11,90]. Cancer therapy-induced metabolic syndrome is a key modifiable driver of CVD and combines with pre-existing CV risk, making cardiotoxicity a major competing cause of morbidity and mortality. Subclinical left ventricular dysfunction, arrhythmias, and endothelial damage may limit patients’ ability to tolerate optimal oncologic therapy.

## 3. Nutritional Status and Energy Requirements

### 3.1. Assessment of Nutritional Risk

Nutritional status is a critical issue and a prognostic indicator for survival and cancer therapy-related toxicities in all cancer patients, but it becomes a priority in LC patients burdened by a high rate of malnutrition with a range between 45% and 60%. In LC patients early screening for malnutrition and sarcopenia is mandatory [23,24,91,92,93]. Malnutrition is present at diagnosis in 35–60% of patients and the percentage increases in advanced stages with a negative impact on survival, treatment response and quality of life [23].

Tools such as nutritional risk score (screening)-2002 (NRS-2002) [94], Patient-Generated Subjective Global Assessment (PG-SGA) [95], Malnutrition Universal Screening Tool (MUST) [96] and Controlling Nutritional Status (CONUT) score [97], complemented by body composition assessment [dual-energy X-ray absorptiometry (DEXA), computed tomography (CT) and bioimpedance analysis (BIA)] [98,99], have been used to identify patients at risk, including cancer patients, but there are a few nutritional indexes specifically targeted to the oncologic population. One of them is the prognostic immune and nutritional index (PINI) that has been identified as a suitable index to predict clinical outcomes in multiple cancers. It is calculated using the following formula: [ALB (g/dL) × 0.9] − [AMC(/μL) × 0.0007] where ALB is albumin (g/dL) and AMC is the absolute monocyte count/μL [100]. The PINI has also been used in a retrospective cohort study that included 522 stage I-IIIA NSCLC patients, mostly of East Asian ethnicity, and in this population it has shown a robust prognostic value [101].

### 3.2. Energy and Protein Requirements

Energy intake should generally be 25–30 kcal/kg/day, with protein at 1.2–1.5 g/kg/day, adjusted for hypercatabolism or sarcopenia [102,103]. In NSCLC patients, adequate nutrition supports cardiac and skeletal muscle function, immune response, and mitochondrial efficiency [104]. In this scenario anabolic resistance, the reduced ability of skeletal muscle tissue to respond to common anabolic stimuli, such as dietary protein and exercise, by increasing muscle protein synthesis rates, plays a key role in cancer-induced muscle wasting. To counteract this condition, resistance exercise training has been suggested for its ability to reduce anabolic resistance through improvements in oxidative metabolism [105] along with dietary essential amino acids [106].

Adequate nutrition supports cardiac and skeletal muscle function, immune response, and mitochondrial efficiency.

### 3.3. Macronutrient Composition and Dietary Patterns

High-quality proteins, complex carbohydrates, and unsaturated fats enhance metabolic flexibility and reduce inflammation [107]. CVD is a common comorbidity in LC patients; therefore, fiber-rich food and high-quality carbohydrates which are associated, in the CARDIA study, with lower left ventricular mass index, improved global longitudinal strain, better left ventricular ejection fraction and improved diastolic function, should be recommended [108], even though the small differences in echocardiographic parameters may lead us to question the power of the evidence [109]. Diets that emphasize the use of fruits, vegetables, wholegrains, healthy fats and lean proteins such as the Dietary Approaches to Stop Hypertension (DASH) and the Mediterranean dietary patterns are indeed broadly considered to be beneficial [110,111]. Mediterranean-style diets improve endothelial function and cardiac efficiency, which is particularly important for patients with elevated cardiovascular risk [112,113]. In a preclinical study with rats, a high-fat diet induced cardiac fibrosis and left ventricular dysfunction through an upregulation of the microRNA expressions of fibrotic markers such as connective tissue growth factor (CTGF), collagen-1α1 (Col1α1), collagen 3α1 (Col3α1), and collagen4α1 (Col4α1) and a concomitant upregulation of the protein levels of CTGF, collagen-II, and collagen-IV [114], thus confirming the deleterious effect of a high-fat diet on cardiac remodeling.

It has been shown that the use of fish oil improves body fat, lean body mass, pain, appetite and quality of life in pancreatic and LC patients with cachexia [115,116]. Metabolomic-based studies have hypothesized that the tryptophan–kynurenine pathway metabolites might explain the beneficial effects of the Mediterranean diet (MD) on CVD [117]. The 2026 Dietary Guidance to improve cardiovascular health summarize the features of a heart-healthy diet in eight items that include the choice of plenty of vegetables and fruits, of wholegrain food, of unsaturated fats and minimally processed food, of a minimum intake of added sugars in beverages and foods and of a reduced amount of sodium and avoidance of alcohol consumption [118].

Regarding nutrition tools to prevent myocardial injury, there are only *preclinical* studies that focus on ICI-induced gut dysbiosis. The first study shows that combining periodic fasting-mimicking diet (FMD) cycles with anti-PD-L1 therapy not only improves efficacy, but reduces the infiltration of CD3 and CD8 T cells into myocardial tissue and diminishes both systemic and myocardial markers of oxidative stress and inflammation. Acting on the gut microbiota, this diet has the potential to limit myocardial injury [119]. The second study shows that microbiota-indole-3-propionic acid-heart axis is the mediator of the protective effect of leflunomide against αPD1-induced cardiotoxicity in mice [120].

### 3.4. Micronutrients

Deficiencies in vitamin D, selenium, zinc, and magnesium impair muscle, immune, and cardiac function [121], and the impact may be even greater in cancer patients. A micronutrient deficiency may indeed be found in LC patients. Correction of these deficiencies should improve both oncologic and cardiovascular outcomes. Unfortunately, there are conflicting data on carotenoids such as β-carotene: they seemed to be related to reduced oxidative stress and inflammation related to smoking exposure, but more recent, randomized trials evaluating vitamin A and β-carotene supplementation have failed to confirm these benefits and, in some populations of smokers, have even been suggested to have potentially harmful effects [31]. The role of vitamin D is also uncertain. Possible anticancer activity has been suggested in experimental models in vitro and in vivo, but this positive action has not been proved to be true in clinical practice [122].

Routine antioxidant supplementation during active therapy is discouraged due to potential interference with treatment efficacy. 

### 3.5. Meal Timing and Chrononutrition

Meal timing and composition are often reported in the literature as “time cues” for the circadian system of animal models, but evidence for food “time cue” is scarce in humans [123]. Aligning meal timing with circadian rhythms improves metabolic processes and reduces cardiometabolic risk, thus conferring a relevant role to the concept of chrononutrition. The combination of MD and meal timing/chrononutrition improves the beneficial effects of the diet alone [124]. Time-restricted eating (TRE) improves quality of life and has a beneficial impact on body mass index, adiposity, glucose regulation and inflammation [125]. Chrononutrition restores circadian synchrony, reduces hyperinsulinemia and increases metabolic resilience [126].

### 3.6. The Role of Trained Immunity

Innate immune cells are key regulators of atherosclerosis, given their capacity of a durable proinflammatory phenotype after external or endogenous inflammatory stimuli (e.g., oxidized LDL or high glucose) [127,128,129]. This long-term enhanced response may be modified by dietary components; at the moment, there are only preclinical studies in mice that have documented an epigenetic reprogramming of myeloid progenitor cells after “intermittent” administration of high-fat diets driven by activation of the NLRP3 inflammasome [130]. According to Lavillegrand et al., the intermittent high-fat diet-induced trained immunity is responsible for the enhanced plaque growth during a second 4-week period of a high-fat diet when compared with the effects of an eight-consecutive-week high-fat diet [131]. As far as clinical data are concerned, it seems that MD may attenuate maladaptive trained immunity and systemic inflammation, primarily through its well-known anti-inflammatory effects, diet-induced modulation of the gut microbiota, and improved immune function [132,133,134].

## 4. Exercise

### 4.1. The Multitargeted Effects of Exercise: From Myokines to Exerkines

Robust data support the importance of exercise in the prevention and treatment of noncommunicable diseases (NCDs), such as CVD, obesity, type 2 diabetes mellitus, cognitive decline and many cancers [135,136]. Aerobic training, encompassing moderate-intensity continuous training and high-intensity interval training (HIIT), enhances mitochondrial biogenesis and oxidative capacity in skeletal muscle and improves myocardial energetics, while resistance training drives a different mitochondrial remodeling with a modest effect on mitochondrial biogenesis, oxidative capacity and myocardial energetics. High-intensity aerobic exercise training has a favorable impact on cardiometabolic health (e.g., insulin sensitivity) [137]. A synergistic effect on mitochondrial remodeling can be obtained, combining resistance and endurance training [138]. Exercise mitigates systemic inflammation [136], improves endothelial function [139] and strengthens cardiac and skeletal muscle [140,141], thus reducing the risk of therapy-induced cardiotoxicity [142,143]. Exerkines are molecules modified in response to acute and chronic exercise that mediate the systemic adaptations to exercise [144]; they are the drivers of the beneficial effects of exercise, given their important signaling effects through endocrine, paracrine and/or autocrine pathways. While muscle-secreted hormones (myokines such as IL-6) were initially considered the main source of signaling activities, now the panel of cytokines has broadened to include exercise-induced humoral factors from the heart (cardiokines), the liver (hepatokines), the white adipose tissue (WAT adipokines), the brown adipose tissue (BAT batokines) and the nervous system (neurokines) [145]. Regarding IL-6, it is necessary to point out the chameleonic feature of this cytokine that is both a proinflammatory cytokine and an exercise-induced myokine [146]. A meta-analysis of 30 randomized controlled trials (RCTs) with 2484 participants across different cancer types found an improvement in VO2 peak, resting diastolic blood pressure and resting heart rate after exercise-based interventions [143]. Preclinical studies have documented a positive impact of low-intensity exercise on ICI-induced dilated cardiomyopathy preventing left ventricular dilatation and fractional shortening decline through a regulatory effect on dysfunctional metabolism and autophagy [147].

Exercise may also increase immunotherapy efficacy by mobilizing natural killer (NK) cells and CD8+ T cells into circulation, thus enhancing IL-15/IL-15Rα signaling, promoting tumor vascular normalization and reprogramming “cold” tumors to “hot” microenvironments [148,149,150]. A systematic review of eight studies with 1172 cancer patients found consistent positive effects of exercise on immune function and treatment outcomes [151].

More specifically, in LC patients, exercise programs have demonstrated improvements in cardiorespiratory fitness (CRF) expressed by VO2 peak, physical function, fatigue and quality of life, with non-significant impact on measures of cardiac remodeling [107,152,153]. Prehabilitation (preoperative exercise) specifically reduces postoperative length of stay and complications, while physical activity has long been considered by the National Comprehensive Cancer Network (NCCN) a Category 1 recommendation for cancer-related fatigue [154]. The NCCN Survivorship Guidelines recommend the **“ABCDEs”** of cardiovascular wellness for all cancer survivors, which include **A**ssessment of CVD risk, **B**lood pressure management, **C**holesterol/**C**igarette cessation, **D**iet/**D**iabetes management, and **E**xercise/**E**chocardiogram [155], while the NCCN NSCLC guidelines specifically address the LC population recommending LC-targeted exercise programs and perioperative pulmonary rehabilitation [156].

### 4.2. Role of Exercise in Cancer Treatment-Induced Metabolic Syndrome (CTMS)

CTMS comprises central obesity, insulin resistance, dyslipidemia, and hypertension, and is diagnosed when three or more criteria are met (increased waist circumference, low HDL-Cholesterol, elevated triglycerides ≥ 1.7 mmol/L, BP ≥ 130/85 mmHg, or fasting glucose ≥ 5.6 mmol/L) [157]. Exercise counteracts CTMS as documented in a 2025 umbrella review of 80 meta-analyses (that included LC patients) in which exercise significantly modulates insulin, insulin-like growth factor (IGF-1), insulin-like growth factor-binding protein-1 (IGFBP-1) and C-reactive protein (CRP) [158]. Favorable results of exercise on CTMS have been extensively documented for breast cancer (BC) survivors: [159,160,161], while LC-specific data on metabolic syndrome are limited [158] even if platinum-based chemotherapy is a recognized contributor of CTMS and that the high prevalence of smoking, obesity and sedentary behavior in LC patients further amplifies the risk of CTMS.

### 4.3. Clinical Implementation and Safety

Individuals living with cancer and cancer survivors are burdened by physical side effects of cancer and its treatment, functional and cognitive impairment, and psychological and economic sequelae [162] with a negative impact on social roles and on quality of life [163]. LC survivors may also have the unpleasant feeling of being stigmatized because of the perception that LC is a self-inflicted disease, and this perception is especially painful for patients who have never smoked [164]. In these patients, rehabilitation and exercise interventions have an extremely favorable role in reducing the negative impact of treatment-related symptoms and in improving quality of life [165]. A meta-analysis of RCT has documented improved cognitive function induced by mind–body exercise (yoga, tai chi, qigong, etc.) in patients with LC [166]. The Guidelines from the American College of Chest Physicians recommend rehabilitation for a better management of persistent cough in lung cancer survivors [167] while The European Respiratory Society/European Society of Thoracic Surgery guidelines endorse rehabilitation for patients at high risk for adverse surgical outcomes [168]. ASCO Guidelines state that “Oncology providers should recommend aerobic and resistance exercise during active treatment with curative intent to mitigate side effects of cancer treatment” noting that “Exercise interventions during active treatment reduce fatigue; preserve cardiorespiratory fitness, physical functioning, and strength”, in some population exercise may even improve quality of life (QoL), and reduce anxiety and depression. All these beneficial effects of exercise during treatment have a low risk of adverse events. The Guidelines also recommend preoperative exercise for patients undergoing surgery for lung cancer to reduce length of hospital stay and postoperative complications [107]. The improvement in cardiorespiratory fitness, muscle strength, quality of life and fatigue can also be obtained in advanced LC as outlined in a recent systematic review and meta-analysis including nine RCTs [152]. As far as physical activity in patients with advanced cancer is concerned, the NCCN Guidelines (Version 4.2026) recommend *consideration* for specific vulnerable populations such as survivors with bone loss or bone metastases [156]. Unfortunately, cancer rehabilitation is underutilized [107,169,170]. To address this care gap, in 2017, the World Health Organization (WHO) has launched “Rehabilitation 2030” to increase global access to rehabilitation as an essential component of healthcare service for individuals with NCD [171], defining oncology a priority area for this program [172].

### 4.4. Barriers/Solutions (Training at Home and Hybrid Training)

A scarce awareness of the positive effects of rehabilitation, especially in the presurgical scenario, plays a relevant role in constraining a full implementation of rehabilitation programs, followed by logistic difficulties, frailties, and digital illiteracy [173,174,175,176,177,178,179]. LC patients face unique barriers to exercise, including dyspnea, reduced pulmonary function, anxiety, depression, insomnia, pain and cancer-related fatigue. These barriers are even more challenging in remote and rural areas. Another issue is also gaining momentum: “financial toxicity” driven by the “chronicity” of cancer and fueled by expensive therapies and continuous medical investigations. Financial toxicity including “intrinsic” factors (e.g., gender, age, ethnicity, and lower income), and “disease-related” factors (e.g., costs of systemic anticancer) has a significant impact not only for healthcare systems but also for the quality of life of cancer patients and their families [180,181,182,183,184]. As highlighted by the ESMO expert consensus statements, socioeconomic determinants play a critical role in this regard [184]. To counteract these barriers there are some innovations for neglected, under-represented patients. A paper by Menezes et al. on technology-based cardiac rehabilitation therapy in women (a well-known under-represented population in cardiac rehabilitation, especially in lower-income settings), has stressed the importance of a tailored, technology-based comprehensive cardiac rehabilitation therapy to improve accessibility and has emphasized the critical role of patient preferences [185]. More specifically, in the oncological setting, a prospective study on a real-world population of 180 cancer patients undergoing oncologic treatment has documented the feasibility, the effectiveness (improvements on six-minute walking test, leg press strength, handgrip strength and flexibility tests) and the safety of a tailored exercise program [186], while a single-blind, 3-arm randomized controlled trial with an 8-week follow-up has documented increased total physical exercise driven by a brief oncologist-delivered recommendation combined with a dedicated guidebook [187]; however, data the on long-term impact of exercise are still needed. A recent cost/effectiveness evaluation of a multimodal prehabilitation program in 284 high-risk LC surgery has documented clinical effectiveness and economic benefit even in this vulnerable cohort of patients [188]. Technology-based, remote, at home or hybrid programs, along with increased awareness on the beneficial effects of rehabilitation, may pave the way for widespread use of rehabilitation programs throughout the cancer journey.

### 4.5. Integration with Nutritional Support

The synergistic combination of exercise and tailored nutritional interventions preserve lean mass, enhances therapy response, and reduces metabolic and cardiac stress. The synergy is explained by the sensitizing effect of exercise on muscle to the anabolic actions of amino acids, thus augmenting muscle protein anabolism compared to each intervention alone, as documented in sarcopenia [189,190].

This multimodal approach addresses both oncologic and cardiovascular outcomes [191] and it has been proven successful in LC patients [192].

### 4.6. Physical Activity and Trained Immunity

Physical activity has a relevant impact on immunity, modulating innate immune function and possibly influencing trained immunity through metabolic and epigenetic mechanisms. While robust evidence has documented the role of regular moderate exercise in enhancing innate immune parameters (e.g., increased activity of NK cells), improving immunosurveillance and reducing systemic inflammation [193,194,195,196,197], the link between physical activity and trained immunity has not been established yet in clinical trials.

## 5. Integrative Approaches and Clinical Evidence

### 5.1. Multimodal Interventions

The NCCN and AHA recommend a multidisciplinary team including cardio-oncologists, exercise oncology specialists, registered dietitians (especially specialists in Oncology Nutrition) and behavioral health professionals to address the multifaceted needs of cancer patients [155,189]. Evidence indicates that combined nutrition and exercise programs improve functional capacity, reduce systemic inflammation, mitigate cancer therapy-induced metabolic syndrome [198] and enhance therapy response while reducing CV risk. Prehabilitation, defined by Silver and Baima as “a process of cancer continuum of care that occurs between the time of cancer diagnosis and the beginning of acute treatment and includes physical and psychological assessments that establish a baseline functional level, identify impairment and provide interventions that promotes physical and psychological health to reduce the incidence and/or the severity of future impairment” [199], may also increase cardiovascular reserve during subsequent cancer therapy [200] and has been proved effective by Voorn et al. [176]. A meta-analysis of 11 studies on prehabilitation in LC patients concluded that moderate-to-intense preoperative exercise has beneficial effects on aerobic capacity, physical fitness, and quality of life in this vulnerable patient population [201]. A more recent study has compared postoperative outcomes in LC patients with respiratory disease, predicted length of stay and neoadjuvant therapy enrolled in a multimodal prehabilitation program versus control (147 matched pairs per group). The control group showed significantly higher rates of overall and major complications whereas patients who underwent multimodal prehabilitation had a significantly lower Comprehensive Complication Index and a reduced intensive care unit admission rate [177].

### 5.2. The Multifaceted Network of Gut Microbiota

The gut microbiota (GM) is a sophisticated ecosystem and a crucial player in physiological homeostasis and systemic health [202]. GM includes bacteria, fungi, viruses and other organisms, and, along with its metabolites, has a pivotal role in many cross-organ networks giving rise to different axes that regulate myocardial health such as the gut–brain–heart, gut–heart–muscle, gut–liver–heart and gut–lung–heart axes [203]. The gut–heart–muscle axis, a complex multidirectional network between the GM, cardiac muscle and skeletal muscle physiology, may lead to HF, muscle decline, and metabolic diseases [204,205,206]. GM and its metabolites activate fibroblasts, but they also have an immunomodulating and anti-inflammatory role. The intestinal wall harbors gut-associated lymphoid tissue and residual macrophages and stimulates dendritic cells, increasing the secretion of IgA and the production of antibacterial protein [207]. GM and microbial metabolism are involved in the development of cardiometabolic disease [208]; moreover, GM has a bidirectional and dynamic interaction with its host’s immune system [209] and with the tumor immune microenvironment, influencing the effectiveness of immunotherapy [210] and the immune-related adverse events [211]. A recent study with a small number of patients (19) has found that ICI treatment seems to be more effective in patients with more indigenous bifidobacteria [212]; the prevalence of *Bifidobacterium* in ICI treatment responders compared to non-responders had been previously documented [213,214], thus implying that the antitumor efficacy may be enhanced by *Bifidobacterium.* The small sample size and the heterogeneity of these studies have led to demand for larger studies with more homogenous populations to eventually confirm the link between GM and immunotherapy outcomes. Antibiotics, too, have a role in ICI efficacy, given their impact on gut microbial dysbiosis [215]. A prior antibiotic treatment reduced the efficacy of nivolumab in a prospective trial of 72 patients [216]; this observation suggests a close association between the gut microbiome and immunomodulation [217]. Another intriguing issue is the role of GM in activating myofibroblast and inducing fibrosis. This effect is intermingled in a complex network of neuromodulation in which sympathetic hyperactivity enhances peripheral inflammation and subsequent fibrosis, whereas cholinergic stimulation seems to have an anti-inflammatory and anti-fibrotic effect [218].

Nutrition has a significant bidirectional relationship with GM; while diet modulates gut microbial composition and function, gut metabolites obtained by dietary substrates affect glucose and lipid metabolism, vascular tome and immune signaling [219,220]. A high-fiber diet increases the diversity of the gut microbiome, the amount of short-chain fatty acids (SCFAs) and of hydrogen sulfide (H2S); SCFAs exert anti-inflammatory effects through G-protein-coupled receptors, eventually reducing hypertension and fibrosis [221,222]. MD, for instance, enhances the growth of beneficial bacteria whose metabolites, including SCFAs, decrease proinflammatory microbial species and reduce metabolic dysfunction. All these effects strengthen gut barrier integrity, reducing intestinal permeability and activation of systemic inflammatory pathways [223,224]. On the other end, Western diets may induce a reduction of microbial variety of genus and phyla, leading to dysbiosis, dysfunction of barrier permeability and irregular stimulation of immune cells, thus increasing the risk of chronic diseases [225]. Moreover, obesity is frequently associated to a leaky gut that enhances adipose tissue inflammation [37].

Physical exercise, too, positively modulates the GM, increasing anti-inflammatory metabolites such as SCFA, which support cardiac and skeletal muscle metabolism and reduce systemic inflammation [226]. Exercise can significantly modify the genes of most Bacteroides and Clostridium species involved in the synthesis of SCFAs. In prediabetic subjects without drug treatment, GM increases its ability to produce SCFAs after exercise [227]. Sample analysis of marathon runners showed a post-race increase in SCFAs [228]. Given the great impact of GM on inflammation and immunity, it is mandatory to develop an effective strategy in LC patients to maintain a healthy gut microenvironment, particularly before and during ICI treatment, even when antibiotics are used.

### 5.3. Personalized Lifestyle Interventions

Precision nutrition and exercise programs tailored to metabolic, genetic, and microbiomic profiles maximize efficacy and adherence. CV monitoring should be integrated to identify early signs of subclinical cardiotoxicity. Digital tools can support ongoing assessment and adaptation of interventions.

## 6. Future Perspectives

As LC survival improves, CVD emerges as a major competing risk. Therapy-induced cardiotoxicity, allostatic load [229,230,231], metabolic stress, and financial toxicity [184] may limit adherence to lifestyle interventions and reduce their effectiveness. Future research should focus on longitudinal studies evaluating integrated nutrition and exercise programs, incorporating cardiovascular endpoints alongside oncologic outcomes. Microbiota composition may become a useful predictor of response to immunotherapy and may be modulated [232]. Trained immunity may gain momentum in the complex interplay of cancer and CVD with more extensive clinical studies. Personalized approaches using biomarkers of metabolism, inflammation and cardiac function, combined with digital health monitoring, may optimize effectiveness while mitigating financial and logistical barriers. Emerging modulators of vascular inflammation and remodeling such as Chemerin Receptor 23 (ChemR23) may be therapeutically targeted in atherosclerosis and CVD [233,234]. New technology-based, remote, at-home or hybrid programs should increase rehabilitation programs throughout the cancer journey [155], in line with the WHO’s project aiming to increase rehabilitation programs [171] and the more ambitious aim of providing more equitable access to prehabilitation services [178], through a wider implementation of telehealth policies for older adults who represent the majority of the LC population [179].

## 7. Conclusions

Malnutrition, sarcopenia, systemic inflammation, cancer therapy-induced metabolic syndrome and elevated CV risk compromise treatment response and survival in LC patients. CVD remains a leading cause of death in cancer survivors, and metabolic syndrome is a key modifiable driver of CV risk. GM is gaining momentum for its role in the delicate balance between microbes and the host immune system, given the fact that dysregulation of this equilibrium leads to chronic low-grade inflammation through an uncontrolled immune activation [235]. Non-pharmacological interventions such as tailored nutrition and structured exercise improve metabolic balance, preserve cardiac and skeletal muscle function, enhance therapy tolerance, and reduce the likelihood of CV events competing with oncologic outcomes. Integration of these interventions into standard care, with attention paid to metabolic, cardiovascular, and socioeconomic factors, represents a critical step toward personalized, cardio-metabolically oriented supportive care, improving both survival and quality of life in LC patients. Counseling on CVD risk factors and the ABCDEs principles of CVD risk assessment where diet and exercise play a relevant role must be implemented to reduce the CVD burden on cancer survivorship, as stated by the NCCN Guidelines Version 2.2026 [155]. Cardio-oncologists have the duties of identifying all the vulnerabilities of LC patients at baseline evaluation in order to tackle all the cardiovascular risk factors and the malnutrition phenotypes with both pharmacological and non-pharmacological therapies. All these different elements are not separated; they are all intertwined and they may eventually meet at a molecular level. This the main message of this narrative review.

## Data Availability

The original contributions presented in the study are included in the article, further inquiries can be directed to the corresponding author.

## Abbreviations

The following abbreviations are used in this manuscript:

AECs	Airborne environmental contaminants
ALK/ROS1	Anaplastic lymphoma kinase/c-ros oncogene 1
AHA	American Heart Association
BAT	Brown adipose tissue
BC	Breast cancer
BIA	Bioimpedance analysis
BP	Blood Pressure
BRAF/MEK V	Raf murine sarcoma viral oncogene homolog B/mitogen-activated extracellular signal-regulated kinase
ChemR23	Chemerin Receptor 23
CKM	Cardiovascular–kidney–metabolic
CONUT	Controlling Nutritional Status
CRF	Cardiorespiratory fitness
CRP	C-reactive protein
CT	Computed tomography
CTGF	Connective tissue growth factor
CTMS	Cancer treatment-induced metabolic syndrome
CV	Cardiovascular
CVD	Cardiovascular disease
DASH	Dietary Approaches to Stop Hypertension
DEXA	Dual-energy X-ray absorptiometry
EC	Endothelial cells
FGF	Fibroblast growth factor
GM	Gut microbiota
HDL	High-density lipoprotein
HF	Heart failure
H2S	Hydrogen sulfide
hs-CRP	High-sensitivity C-reactive protein
ICIs	Immune checkpoint inhibitors
IL	Interleukin
IGF	Insulin-like growth factor
IGFBP	Insulin-like growth factor-binding protein
irAEs	Immune-related adverse events
LC	Lung cancer
LDL	Low-density lipoprotein
LRR	[leucine-rich repeat]-containing
MD	Mediterranean diet
MMP	Matrix metalloproteinase
mtDNA	Mitochondrial DNA
MUST	Malnutrition Universal Screening Tool
NCCN	National Comprehensive Cancer Network
NK	Natural killer
NLRP3 NOD	[Nucleotide oligomerization domain]-containing, LRR [leucine-rich repeat]-containing, and PYD [pyrin domain]-containing protein3
NRS-2002	Nutritional risk score (screening)-2002
PDGF	Platelet-derived growth factor
PG-SGA	Patient-Generated Subjective Global Assessment
RCTs	Randomized controlled trials (RCTs)
RET	Rearranged during Transfection
SCFA	Short-chain fatty acids
SDOH	Social determinants of health
TGF-b	Transforming growth factor beta
TRE	Time-restricted eating
VEGF	Vascular endothelial growth factor
WAT	White adipose tissue
